# High-throughput analysis of DNA repair in microplates towards identification of inhibitors

**DOI:** 10.1186/s41021-020-00153-3

**Published:** 2020-03-09

**Authors:** Hiroyuki Kamiya

**Affiliations:** Graduate School of Biomedical and Health Sciences, Hiroshima University, 1-2-3 Kasumi, Minami-ku, Hiroshima, 734-8553 Japan

**Keywords:** DNA repair, Base excision repair, High-throughput analysis

## Abstract

Environmental factors can inhibit DNA repair and cause indirect mutagenic actions. Here the author introduces a recent paper on a novel high-throughput system to analyze the various enzymatic activities involved in base excision repair. Such systems will facilitate the identification of compounds that suppressively affect DNA repair.

DNA, RNA, and their precursors (nucleoside 5′-triphosphates) are continuously damaged by environmental and endogenous mutagens/carcinogens, such as alkylating reagents, ultraviolet light, and reactive oxygen species [1–5]. Organisms have developed countermeasures against the lesions, including DNA repair and nucleotide pool sanitization systems. In the case of DNA, unrepaired lesions induce genomic instability and contribute to carcinogenesis.

Environmental factors possibly play two roles in mutagenesis. One is the modification of DNA and 2′-deoxyribonucleotides as described, and the other is the inhibition of DNA repair and nucleotide pool sanitization. Assessing the effects of environmental factors and determining their mechanisms are important to understand the interactions between genes and the environment, and to regulate compounds that are toxic for genetic information.

The base excision repair (BER) system repairs various forms of relatively small base damage, as well as abasic sites and single strand breaks [6]. In the case of damaged base repair, a DNA glycosylase specific for the modified base first recognizes the lesion and cleaves the *N-*glycosyl bond. Monofunctional DNA glycosylases leave an abasic site, and APE1 nicks the DNA strand bearing the abasic site. Bifunctional DNA glycosylase/lyases excise the *N-*glycosyl bond and conduct the strand scission. The strand cleavage and removal of the sugar moiety result in gap formation. Gap-filling by DNA polymerase (pol) and the complete chemical bond formation by DNA ligase restore the original genetic information.

Recently, Healing et al. reported a novel high-throughput system to analyze the various enzymatic activities involved in BER [7]. The assay can monitor the activities at each step in microplates. An oligodeoxyribonucleotide complex is fixed to the surface of a microplate and used as the substrate in the assay (Fig. 1). The complex contains a single lesion (a damaged base, abasic site, gap, or nick) and is labeled with fluorescein at the 5′-end. When uracil or alkyladnine DNA glycosylase removes its damaged base substrate, subsequent alkaline denaturation cleaves the DNA strand and separates the labeled and immobilized strands. Thus, the glycosylase activity can be measured by the disappearance of fluorescein from the well. AP endonuclease activity is also measurable by similar procedures. For the DNA pol (gap-filling) and ligase analyses, the fluorescein retained in the well indicates the activities (an excess amount of T4 DNA ligase is added in the reaction mixture for the pol activity analysis).

**Fig. 1:**
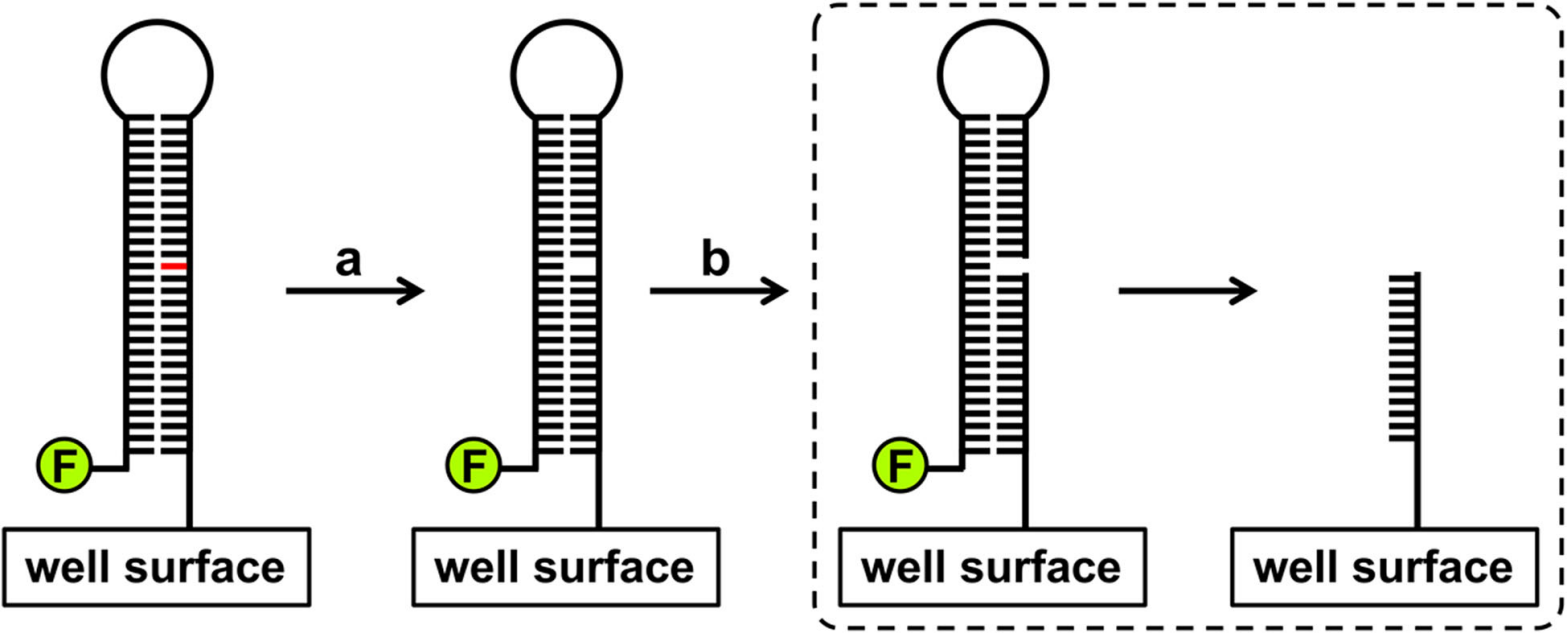
The principle of the uracil DNA glycosylase assay. The substrate oligodeoxyribonucleotide is fixed to the surface of a microplate and bears a uracil base (shown in red) and fluorescein (shown as F) at the 5′-end. The action of glycosylase **a** forms an abasic site, and subsequent alkaline denaturation **b** induces the backbone cleavage and loss of fluorescein

This assay allows evaluations of the inhibitory (and stimulatory) influences of various compounds on the BER system. Many assay methods for BER enzymes have been proposed, and high-throughput screening systems have been used for the identification of DNA glycosylase inhibitors [8–10]. The new assay has a few distinct advantages over the previous assays: it can quantify each step of BER by employing basically the same system and also quantitate overall repair (from the abasic site) across multiple steps of BER. Similar assay systems could be prepared for the nucleotide excision repair. High-throughput analyses using the systems would contribute to the discovery of the indirect mutagenic actions of environmental factors. In addition, analyses of the nuclear extracts from cells treated with compounds would lead to the identification of those that promote the degradation of DNA repair factors [11] and suppress their expression, as well as the inhibitors of each reaction. Conversely, these compounds are double-edged swords and possible therapeutic candidates that could enhance the efficacy of DNA-damaging anticancer drugs. The high-throughput systems will make significant contributions to human health through these two points of action.

## Data Availability

Not applicable.

## Abbreviations

BER: base excision repair
pol: polymerase
